# Synergistic horizontal transfer of antibiotic resistance genes and transposons in the infant gut microbial genome

**DOI:** 10.1128/msphere.00608-23

**Published:** 2023-12-19

**Authors:** Yanwen Ding, Xin Jiang, Jiacheng Wu, Yihui Wang, Lanlan Zhao, Yingmiao Pan, Yaxuan Xi, Guoping Zhao, Ziyun Li, Lei Zhang

**Affiliations:** 1 Microbiome-X, School of Public Health, Cheeloo College of Medicine, Shandong University, Jinan, China; 2 Shandong University, State Key Laboratory of Microbial Technology, Qingdao, China; 3 University of Chinese Academy of Sciences, Chinese Academy of Sciences, CAS Key Laboratory of Computational Biology, Bio-Med Big Data Center, Shanghai Institute of Nutrition and Health, China National Institute of Health, Shanghai, China; University of Kentucky College of Medicine, Lexington, Kentucky, USA

**Keywords:** association rules mining, antibiotic resistance genes, transposons, infant gut microbiota, horizontal gene transfer

## Abstract

Transposons, plasmids, bacteriophages, and other mobile genetic elements facilitate horizontal gene transfer in the gut microbiota, allowing some pathogenic bacteria to acquire antibiotic resistance genes (ARGs). Currently, the relationship between specific ARGs and specific transposons in the comprehensive infant gut microbiome has not been elucidated. In this study, ARGs and transposons were annotated from the Unified Human Gastrointestinal Genome (UHGG) and the Early-Life Gut Genomes (ELGG). Association rules mining was used to explore the association between specific ARGs and specific transposons in UHGG, and the robustness of the association rules was validated using the external database in ELGG. Our results suggested that ARGs and transposons were more likely to be relevant in infant gut microbiota compared to adult gut microbiota, and nine robust association rules were identified, among which *Klebsiella pneumoniae*, *Enterobacter hormaechei_A*, and *Escherichia coli_D* played important roles in this association phenomenon. The emphasis of this study is to investigate the synergistic transfer of specific ARGs and specific transposons in the infant gut microbiota, which can contribute to the study of microbial pathogenesis and the ARG dissemination dynamics.

**IMPORTANCE:**

The transfer of transposons carrying antibiotic resistance genes (ARGs) among microorganisms accelerates antibiotic resistance dissemination among infant gut microbiota. Nonetheless, it is unclear what the relationship between specific ARGs and specific transposons within the infant gut microbiota. *K. pneumoniae*, *E. hormaechei_A*, and *E. coli_D* were identified as key players in the nine robust association rules we discovered. Meanwhile, we found that infant gut microorganisms were more susceptible to horizontal gene transfer events about specific ARGs and specific transposons than adult gut microorganisms. These discoveries could enhance the understanding of microbial pathogenesis and the ARG dissemination dynamics within the infant gut microbiota.

## INTRODUCTION

Antibiotic resistance (AR) poses a significant and growing threat to global health and economic well-being. The number of deaths associated with AR in 2019 is estimated at 4.95 million, of which direct resistance deaths could reach 1.27 million (1). Of these, the major pathogens associated with AR-related deaths (*E. coli*, *Staphylococcus aureus*, *K. pneumoniae*, *Streptococcus pneumoniae*, *Acinetobacter baumannii*, and *Pseudomonas aeruginosa*) caused 929,000 deaths (1). Not only that, according to the U.S. Centers for Disease Control and Prevention, the mortality rate of methicillin-resistant *S. aureus* infection has surpassed that of acquired immune deficiency syndrome, Parkinson’s disease, and murder (2). The World Health Organization has highlighted that if AR continues at its current rate, it could kill 10 million people a year by 2050 (3). The AR dissemination is a cause for concern, and how to mitigate it has become a hot topic of research.

The intestine is rich in antibiotic resistance genes (ARGs) and has been shown to be an important site for AR dissemination (4, 5). Of concern was that infants have a higher abundance of ARGs in their gut flora relative to adults (6, 7), and antibiotic exposure during this period can adversely affect long-term health by disrupting gut microbial maturation over time (7). It is estimated that 80% of children in high-income countries receive antibiotics in the first 48 months of life (7, 8), and the rate is worse in low- and middle-income countries, where each child will receive an average of 11 antibiotic courses in the first two years (7, 9). Therefore, overuse of antibiotics accelerates the spread of AR among bacteria and poses serious health problems (7). Antibiotic exposure in infancy is strongly associated with childhood asthma, allergies, respiratory disorders, and attention-deficit/hyperactivity disorder (ADHD) (10–12), with 20%–40% of affected infants found to be using antibiotics inappropriately (13). Notably, colonization by multi-drug-resistant (MDR) bacteria is a precursor to invasive infections such as sepsis, and in particular, neonatal sepsis (NSS) can cause a serious disease burden in low- and middle-income countries (14). After antibiotic treatments, the composition and diversity of intestinal flora change significantly in infants, with a decrease in the abundance of *Bifidobacterium* spp. and an increase in the abundance of *Klebsiella* spp. and *Enterococcus* spp. (15). Meanwhile, amoxicillin and azithromycin, considered the most commonly used antibiotics in infants, significantly reduce intestinal flora diversity (16). Of note, the chloramphenicol resistance gene is detected in gut microorganisms in 0- to 2-year-old infants who had never used antibiotics, and there are resistance genes that are resistant to the last line of defense against antibiotics, such as colistin and tigecycline in infant gut microbiota (17, 18).

The widespread transfer of mobile genetic elements (MGEs) carrying ARGs among microorganisms accelerates the AR dissemination among infant gut microbiota (19). It is of interest that, despite the prohibition of tetracycline during pregnancy and in children under eight years of age (20), a large number of tetracycline resistance genes have been detected in infants and maternal feces, highlighting the transfer of ARG between the environment and the human gut (7, 20, 21). Recent studies have reported that MGEs can be involved in maternal shaping of the infant’s gut flora as an important mediator of horizontal gene transfer (HGT) (22); this follows a study that found a higher abundance of MGEs in infant gut microorganisms than in mothers (6). Notably, with little or no antibiotic use in newborns, 6.4% of ARGs are syntenic or located in proximity to MGEs, usually transposons or integrases (18). However, such studies currently focus on small-sample metagenomic next-generation sequencing data that does not provide a comprehensive picture of the infant gut microbial genome, while the relationship between specific ARGs and specific MGEs has not been elucidated.

In this study, association rules mining is used to discover association relationships in transactional databases, and there are different application scenarios, such as Chinese medicine prescription data (23) and disease clinical symptom data (24). Similarly, there are wide applications in the field of bioinformatics (25), such as probing gene expression patterns in specific cells (26), identifying associated genes in gene expression and methylation data (27), and cancer co-expression gene module detection (28). Here, we use the Unified Human Gastrointestinal Genome (UHGG) reference database (29) and the Early-Life Gut Genomes (ELGG) reference database (30) to comprehensively characterize the real infant gut microbial genome. Due to the inability of integrases to move autonomously during HGT events (31), while phage databases are incomplete (32), and plasmids with autonomously replicating structures are lost in large numbers in binning (33, 34), we focus on the relationship between the horizontal transfer of ARGs and transposons in the infant gut microbial genome. Based on association rules mining (26), we identified nine pairs of robust association rules for specific ARGs and specific transposons and determined the species origin of these nine robust association rules, while we found that specific ARGs and specific transposons were more likely to be relevant in infant gut microbiota compared to adult gut microbiota.

## RESULTS

### ARGs and transposons were more likely to be relevant in infant gut microbiota compared to adult gut microbiota

To facilitate a mutual comparison of gut microbial genomes between infants (0–3 years) and adults (19–65 years) as controls, we controlled in UHGG that the data from both genomes originated from the same countries and met quality filtering criteria (Table S1). Consequently, 7,727 and 24,165 MAGs were included in the infant and adult gut microbial genomes, respectively. After gene annotation, we found that the infant gut microbial genome was smaller than the adult gut microbial genomes; however, more ARGs and transposons were annotated, specifically 52,585 ARGs and 4,626 transposons in the infant gut microbial genome. These findings suggested a higher susceptibility of the infant gut microbiota to synergistic horizontal transfer of ARGs and transposons compared to adult gut microbiota.

**FIG 1:**
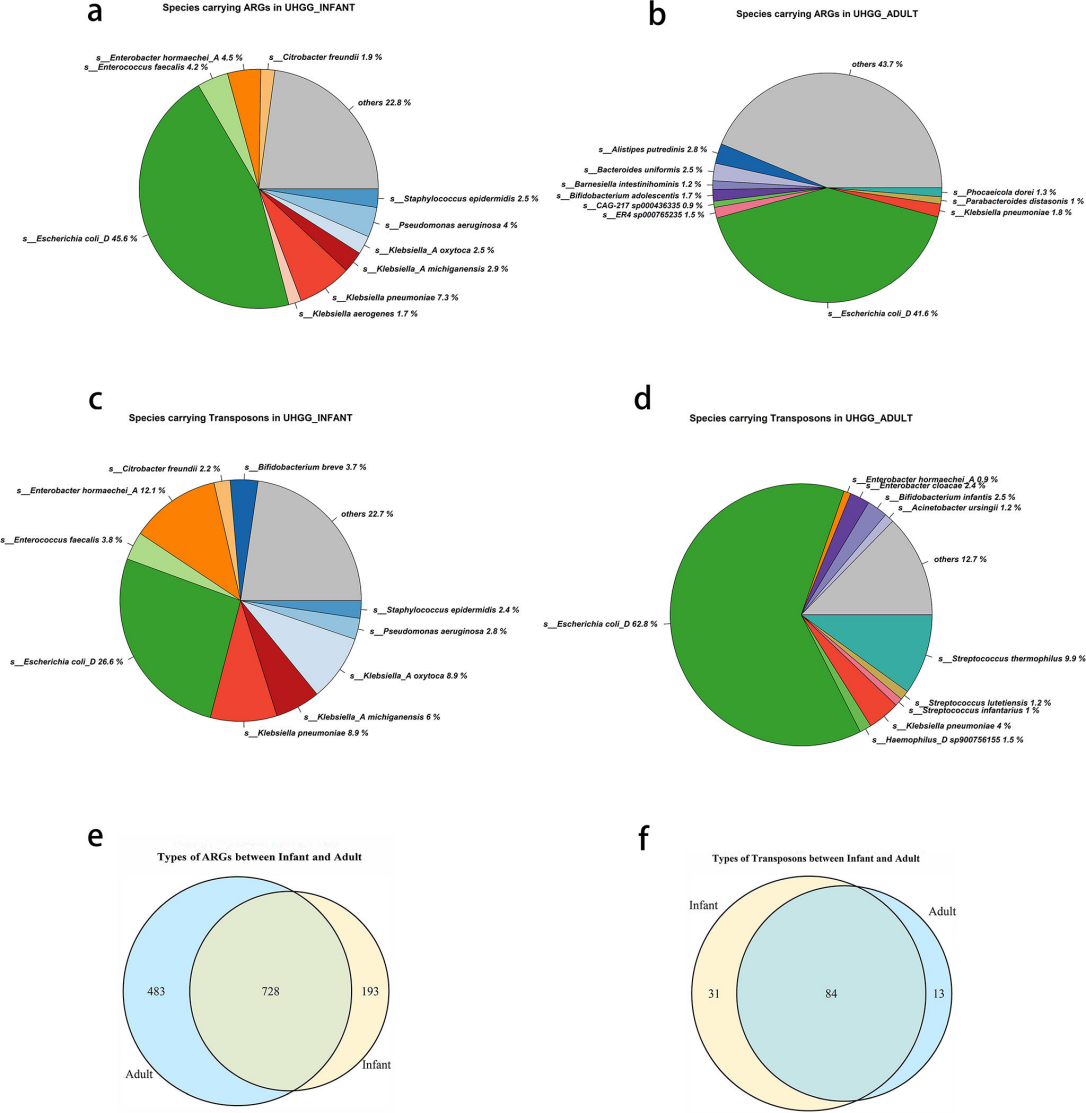
Types of species, ARGs and transposons between infant and adult. (a) Species carrying ARGs in infant. (b) Species carrying ARGs in adult. (c) Species carrying transposons in infant. (d) Species carrying transposons in adult. (e) Types of ARGs between infant and adult. (f) Types of transposons between infant and adult.

The results of age-specific ARGs and transposons annotation are shown in Fig. 1. *E. coli_D* and *K. pneumoniae* were primary species that carried ARGs in infant gut microbiota; meanwhile, *E. coli_D* and *Alistipes putredinis* were primary species that carried ARGs in adult gut microbiota (Fig. 1a and b). *E. coli_D* and *E. hormaechei_A* were the major species that carried transposons in the infant gut microbiota, while *E. coli_D* and *Streptococcus thermophilus* carried the most part of transposons in the adult gut microbiota (Fig. 1c and d). Moreover, 728 types of ARGs and 84 types of transposons were found in both infant and adult gut microbiota (Fig. 1e and f).

**FIG 2:**
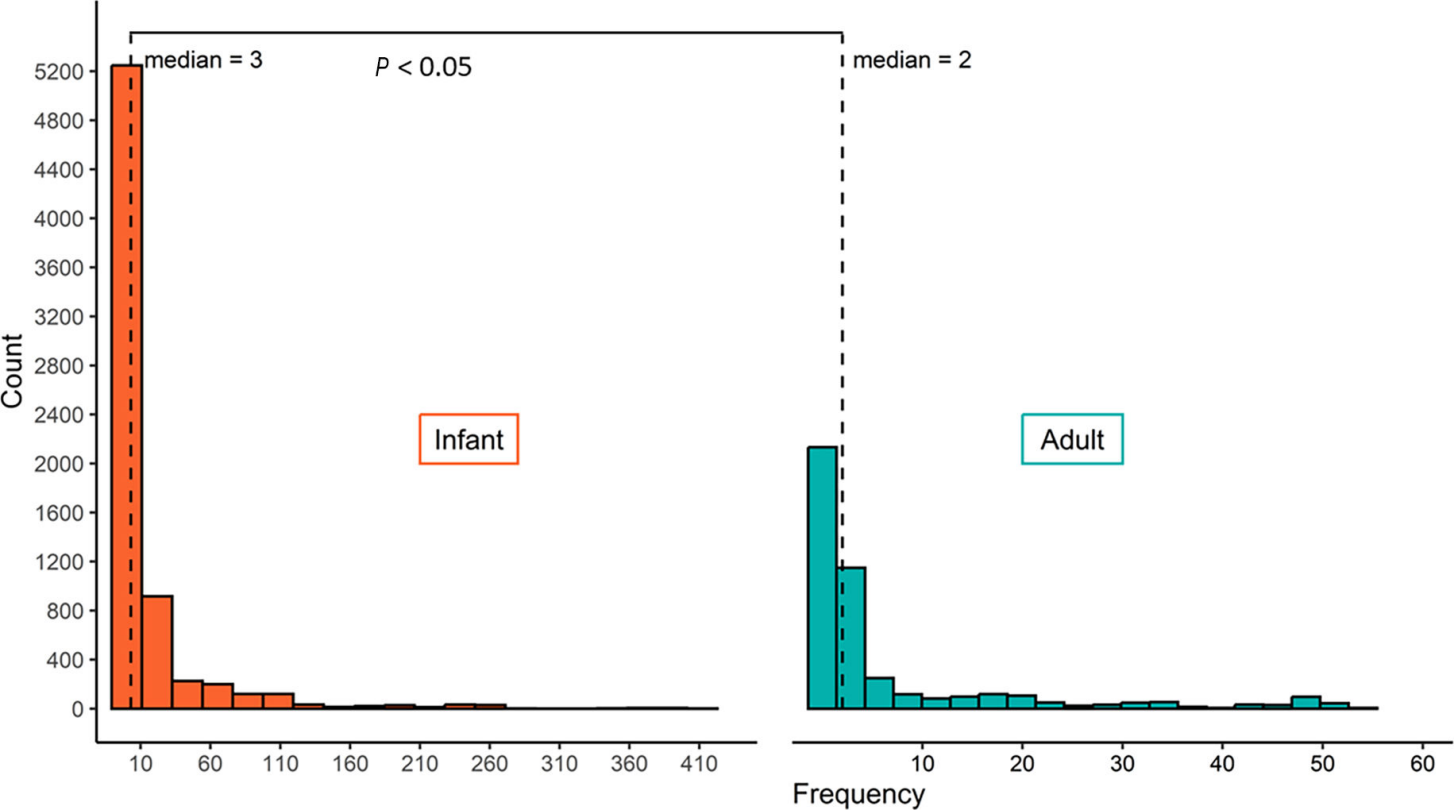
The frequency distribution histogram of MAGs that carry statistically significant specific ARGs and specific transposons.

To statistically test whether infant gut microbiota is more susceptible to occur in synergistic horizontal transfer than adult gut microbiota, we performed a Wilcoxon rank sum test for the count of MAGs with both statistically non-independent specific ARGs and specific transposons between infant and adult. The results showed that infant gut microorganisms were more susceptible to HGT events than adult gut microorganisms (Fig. 2, *P* < 0.05). Meanwhile, these findings highlighted the importance of focusing on the association rules that reflect patterns where transposons and ARGs occur simultaneously in the infant gut microbiota. Moreover, in the exploration of association rules, the aim is to further identify robust association rules.

### Nine robust association rules of specific ARGs and specific transposons were identified in the infant gut microbial genome

To identify association rules for specific ARGs and specific transposons in the infant gut microbial genome, we performed association rules mining on the filtered data from UHGG (Table S1) and initially identified 19 association rules (Table S2, with a chi-square test *P*-value less than 0.05, support greater than 0.01 and effective rate greater than 0.06).

**FIG 3:**
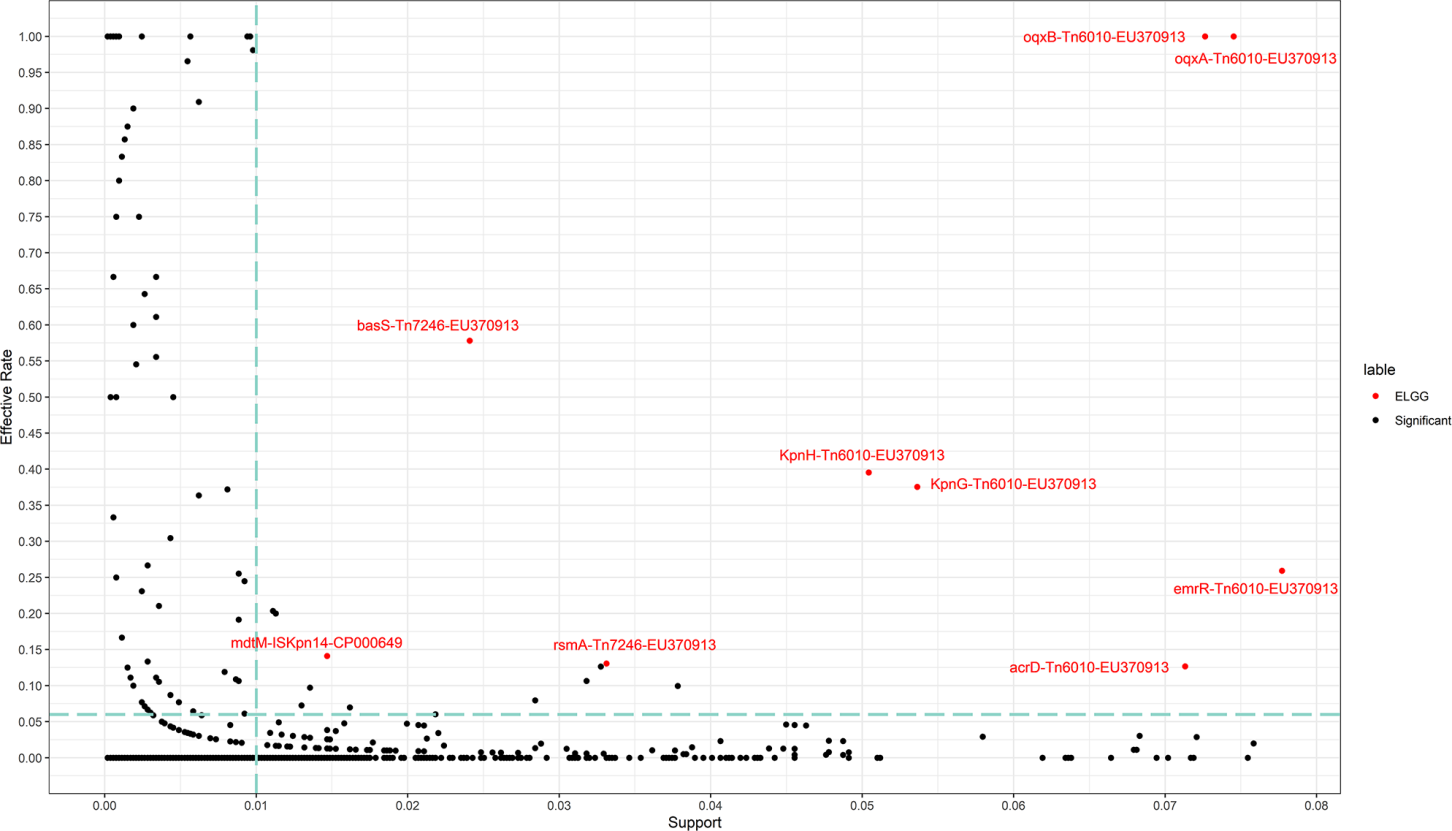
Scatter plot of the results of association rules mining in the UHGG_INFANT and ELGG databases. The black dot represents results of association rules mining in the UHGG_INFANT database. The vertical dotted line represents the standard two about the support should be larger than 0.01. The horizontal dotted line represents the standard of three about the effective rate should be larger than 0.06. The red dot represents the results of robust association rules that are verified by the ELGG database, which is the external database.

Robust association rules are those that are still identified after validation of association rules mining with the external data set from ELGG. Next, nine robust association rules were identified in this study (Table 1; Fig. 3). Considering these results, Tn6010-EU370913 was the important transposon that was associated with many ARGs, such as *oqxA*, *oqxB*, *kpnH*, *kpnG*, *emrR*, and *acrD*. In addition to *acrD* that had resistance to aminoglycoside antibiotic, other ARGs could show resistance to fluoroquinolone antibiotic. Of note, the *oqxA* and *oqxB* were 100% effective with Tn6010-EU370913, a result reported in a previous study (35), which indicates that the analysis process of this study was valid. Meanwhile, Tn7246-EU370913 was often associated with *basS* and *rsmA*. The *basS* had resistance to peptide antibiotic, and *rsmA* had resistance to fluoroquinolone, diaminopyrimidine, and phenicol antibiotic. Furthermore, ISKpn14-CP000649 was associated with *mdtM*, which had resistance to many kinds of antibiotics.

**TABLE 1:** Nine robust association rules of specific ARGs and specific transposons

ARGs	Transposons	Support	Effective rate
*oqxA*	Tn6010-EU370913	0.07	1.00
*oqxB*	Tn6010-EU370913	0.07	1.00
*basS*	Tn7246-EU370913	0.02	0.58
*K. pneumoniae KpnH*	Tn6010-EU370913	0.05	0.40
*K. pneumoniae KpnG*	Tn6010-EU370913	0.05	0.38
*emrR*	Tn6010-EU370913	0.08	0.26
*mdtM*	ISKpn14-CP000649	0.01	0.14
*rsmA*	Tn7246-EU370913	0.03	0.13
*acrD*	Tn6010-EU370913	0.07	0.13

### *K. pneumoniae*, *E.hormaechei_A*, and *E. coli_D* played important roles in the synergistic horizontal transfer of ARGs and transposons in infant gut microbiota

**FIG 4:**
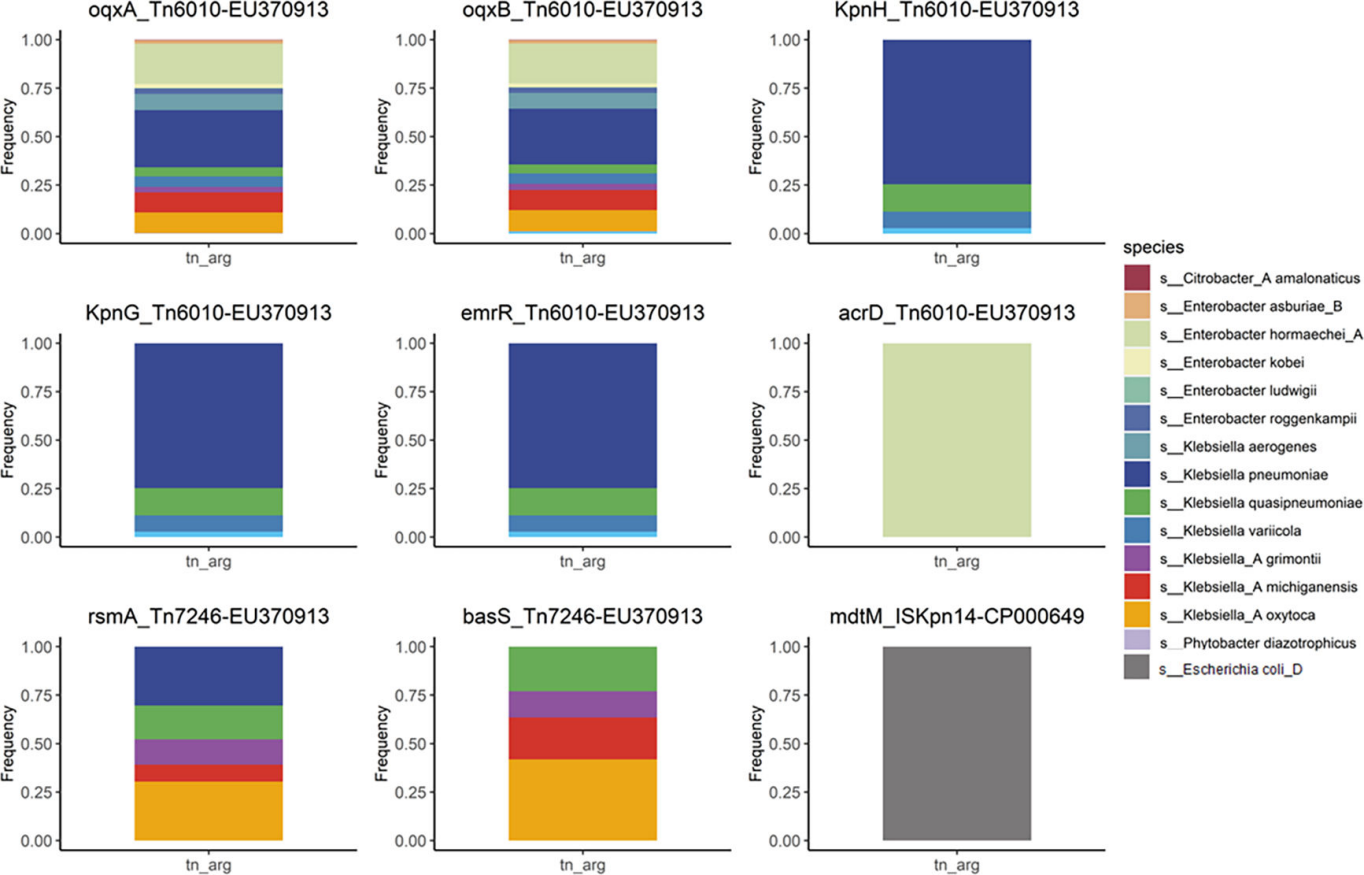
The proportion of species that the specific ARGs and the specific transposons at the same contig in nine robust association rules.

Figure 4 shows the percentage of species in which specific ARGs and specific transposons appeared in the same fragment for the nine robust association rules described above. The results showed that *acrD* and Tn6010-EU370913 appeared in the same allele for species, both from *E. hormaechei_A*, while *mdtM* and ISKpn14-CP000649 appeared in the same allele for species, both from *E. coli_D*. Notably, we found that *K. pneumoniae* was the major species source for the six robust association rules (Fig. 4), with the specific proportions shown in Table 2. We also found that the ARGs for these six robust association rules could show resistance to fluoroquinolone antibiotics. In conclusion, some robust association rules might have species specificity that meant different association rules had different major species, such as *acrD* and *mdtM*. Meanwhile, the horizontal transfer of Tn6010-EU370913 and Tn7246-EU370913 might accelerate the resistance of *K. pneumoniae* to fluoroquinolone antibiotics.

**TABLE 2:** *K. pneumoniae* percentage of six robust association rules

Association rules	Counts^*a*^	Rate^*b*^ (%)
*oqxA* and Tn6010-EU370913	386	29.50
*oqxB* and Tn6010-EU370913	396	28.80
*K. pneumoniae kpnH* and Tn6010-EU370913	106	74.50
*K. pneumoniae* kpnG and Tn6010-EU370913	107	74.80
*emrR* and Tn6010-EU370913	107	74.80
*rsmA* and Tn7246-EU370913	23	30.40

^*a*^ The count of MAGs that the specific ARGs and specific transposons appeared in the same contig.

^*b*^ The *K. pneumoniae* percentage of a.

## DISCUSSION

Several studies have reported that transposons are involved in the dissemination of ARGs in infant gut microbiota, but the specific nature of this relationship has not been fully elucidated (18, 19, 36, 37). Meanwhile, small-sample metagenomic next-generation sequencing data limits the findings of the relationship between specific ARGs and specific transposons. Consequently, our study provided new insight into the horizontal transfer relationships between specific ARGs and specific transposons in the infant gut microbiome genome. We found that specific ARGs and specific transposons were more likely to be relevant in infant gut microbiota compared to adult gut microbiota, which partly explained why the abundance of ARGs in infant gut microbiota was higher than that in adult (6, 7). It might be the basis for the continuous increase of some ARGs in infant gut microbiota. Thus, our findings combined with those of an earlier study (38) could provide corresponding theoretical support for the lateral transfer potential of MGEs in the infant gut microbiota. In this analysis, we identified nine robust association rules, with a total of three types of transposons, each of them could carry multiple ARGs for horizontal transfer, which also could accelerate the spread of ARGs in infant gut microbiota. These three transposons come from the compound family and IS1 family, among which IS1 family members are more likely to carry ARGs, which has been reported (39). Regarding the compound family, Tn6010-EU370913 and Tn7246-EU370913 were found to have robust association rules with several ARGs. Tn6010-EU370913 and Tn7246-EU370913 have conserved insertion sequences, IS26.var1 and IS1R, at both the head and tail ends, which are important for the horizontal transfer of ARGs. The special structure of compound transposons provides advantages for modifying them to alleviate AR in infant gut microbiota (40).

*K. pneumoniae* is a clinically important bacteria resistant to multiple antibiotics (36, 41), and our analysis found that *K. pneumoniae* played a key role in the horizontal transfer of ARGs. Experimental studies have reported that Tn1721 carries genes that have resistance to β-lactamase antibiotics in *K. pneumoniae* through the IncFII plasmid (36). Meanwhile, frequent transposition of Tn4401 can accelerate the spread of carbapenem antibiotic resistance in *K. pneumoniae* (37). Whether the transposons Tn6010-EU370913 calculated in our study carrying fluoroquinolone ARGs accelerate the spread of *K. pneumoniae* resistance to fluoroquinolone antibiotics remains to be further verified by experiments. Aminoglycoside antibiotics are the main antibiotics used in the infection of *E. hormaechei* (42). Meanwhile, our analysis also suggested that *acrD* carried by Tn6010-EU370913 accelerates the spread of resistance to aminoglycoside antibiotics in *E. hormaechei_A*. The *mdtM* gene encodes a single polypeptide with a length of 410 amino acids, which is a universal member of the major facilitator superfamily, and a multidrug efflux protein of *E. coli* (43, 44). Our study found that *mdtM* and ISKpn-CP000649 were the robust association rules, which might provide partly theoretical support for the resource of a large number of multidrug efflux proteins in *E. coli*.

Our research has several strengths. Two microbial reference databases provide a powerful condition for our research to reflect the true situation of infant and adult gut microbiota. Meanwhile, association rules mining provides an effective way for our study to explore specific ARGs and specific transposon patterns. However, our study is not without limitations. Firstly, we cannot quantify the expression of ARGs or transposons, because the data are from reference databases. So, their quantitative relationship cannot be calculated. Then, the lack of clinical medication and lifestyle information prevents us from exploring the factors influencing the strength of association rules.

In summary, we identified nine robust association rules from two main microbial reference databases and species, whereby specific ARGs and specific transposons tended to have synergistic horizontal transfer. We also found that specific ARGs and specific transposons were more likely to be associated with infant gut microbiota compared to adult gut microbiota. Based on these findings, we partly provided the dissemination regularity of ARGs and transposons in infant gut microbiota and partly contributed to studies for mitigating the spread of AR in infant gut microbiota.

## MATERIALS AND METHODS

### Data sources and quality control

In this study, we collected two reference databases on human gut microorganisms: the UHGG (29) and the ELGG (30) databases. The UHGG database is the most comprehensive sequence resource for human gut microorganisms to date, with a compilation of 204,938 genomes and 170,602,708 genes analyzed from infant and adult gut microbial data sets from 31 countries across six continents (Africa, Asia, Europe, North America, South America, and Oceania) (29). The ELGG database catalog is the first large-scale catalog of metagenome-assembled genomes (MAGs) designed for the gut microbiome of infants aged 0–3 years, containing 32,277 genomes and 86,678,654 genes from 11 countries across four continents (Asia, Europe, North America, and Oceania). MAGs from UHGG and ELGG that met completeness >50% and contamination <5%, along with a genome quality score (defined as completeness-5 × contamination, QS) >50 were included in this study (45). In parallel, we selected 7,727 infant MAGs (UHGG_INFANT database, 0–3 years) and 24165 adult MAGs (UHGG_ADULT database, 19–65 years) from the UHGG database. UHGG_INFANT and UHGG_ADULT databases were from the same countries: Bangladesh, El Salvador, Fiji, Italy, Peru, Sweden, and United States (Table S1). In addition, a total of 12,471 MAGs from seven countries other than those mentioned above were selected as the external validation database for this study from the ELGG database, with the external validation database coming from seven countries: Estonia, Finland, Luxembourg, New Zealand, Russia, Singapore, and the UK (Table S1).

### Bioinformatics analysis

Functional annotation of ARGs used the Comprehensive Antibiotic Resistance Database (CARD) for MAGs in the UHGG_INFANT, UHGG_ADULT, and external validation databases (46). The parameter input_type selected contig and the parameter alignment_tool selected BLAST. Moreover, we used the TnCentral (35) to annotate transposon genes in the UHGG_INFANT, UHGG_ADULT, and external validation databases, and BLASTN was used for sequence alignment with parameters evalue set to e-5. Identity is set to 80%, and coverage is set to 60% (47).

### Statistical analysis

To find the association between specific ARGs and specific transposons, we chose the association rules mining method. To accurately determine the association rules of specific ARGs and specific transposons, we chose the following three standards, while the following definitions need to be specified in order to accurately describe these three standards, as shown in Table 3.

**TABLE 3:** Symbol description of association rules mining

Value	Description
Xi	Antibiotic resistance gene i
Yj	Transposon j
Xi0Yj0	The count of MAGs that exclude gene Xi and gene Yj
Xi0Yj1	The count of MAGs that exclude gene Xi but include gene Yj
Xi1Yj0	The count of MAGs that include gene Xi but exclude gene Yj
Xi1Yj1	The count of MAGs that include gene Xi and gene Yj
ContigXi1Yj1	The count of MAGs that include gene Xi and gene Yj which are located in the same contig

### Standard 1

The test for statistical independence of specific ARGs and specific transposons was performed using the chi-square test, and the *P*-value of the chi-square test was used to assess the independence of the two genes (48) at a significance test level of α = 0.05.

### Standard 2

Support was used to determine the frequency of MAGs with a specific ARG and a specific transposon occurring simultaneously, as specified in the equation below, with a threshold value of 0.01.


$$Support=\frac{X_{i1}Y_{j1}}{{X_{i1}Y_{j1}+X}_{i0}Y_{j1}+X_{i1}Y_{j0}{+X}_{i0}Y_{j0}}$$

### Standard 3

The efficiency rate was used to determine the ratio of the count of MAGs that include simultaneously a specific ARG and a specific transposon, which are located in the same contig. The definition of the effective rate is specified in the equation below, taking a threshold value of 0.06 (18).


$$Effective\;rate=\frac{Contig_{X_{i1}Y_{j1}}}{X_{i1}Y_{j1}}$$

Statistical analysis of annotation results for ARGs and transposons from two reference databases was performed using R (version 4.1.3), and images in the text were plotted by ggplot2 (version 3.4.2) if not otherwise indicated.

## SUPPLEMENTAL MATERIAL

Table S1 msphere.00608-23-s0001.xlsx The distribution of country in UHGG_INFANT, UHGG_ADULT, and ELGG databases.

Table S2 msphere.00608-23-s0002.xlsx Association rules of specific ARGs and specific transposons in UHGG_INFANT database.

ASM does not own the copyrights to Supplemental Material that may be linked to, or accessed through, an article. The authors have granted ASM a non-exclusive, world-wide license to publish the Supplemental Material files. Please contact the corresponding author directly for reuse.
